# Neuromuscular assessment of force development, postural, and gait performance under cognitive-motor dual-tasking in healthy older adults and early Parkinson's disease patients: Study protocol for a cross-sectional Mobile Brain/Body Imaging (MoBI) study

**DOI:** 10.12688/openreseurope.15781.1

**Published:** 2023-04-17

**Authors:** Uros Marusic, Manca Peskar, Maja Maša Šömen, Miloš Kalc, Ales Holobar, Klaus Gramann, Bettina Wollesen, Anna Wunderlich, Christoph Michel, Aleksandar Miladinović, Mauro Catalan, Alex Buoite Stella, Milos Ajcevic, Paolo Manganotti

**Affiliations:** 1 Science and Research Centre Koper, Institute for Kinesiology Research, Koper, Slovenia; 2 Department of Health Sciences, Alma Mater Europaea Evropski Center Maribor, Maribor, Slovenia; 3 Department of Psychology and Ergonomics, Faculty V: Mechanical Engineering and Transport Systems, Technische Universitat Berlin, Berlin, Berlin, Germany; 4 Faculty of Arts, University of Ljubljana, Ljubljana, Slovenia; 5 Faculty of Medicine, University of Maribor, Maribor, Slovenia; 6 Faculty of Electrical Engineering and Computer Science, University of Maribor, Maribor, Slovenia; 7 Institute of Human Movement Science, Faculty of Psychology and Human Movement, University Hamburg, Hamburg, Germany; 8 Functional Brain Mapping Lab, Department of Basic Neurosciences, University of Geneva, Geneva, Switzerland; 9 Institute for Maternal and Child Health Burlo Garofolo (IRCCS), University of Trieste, Trieste, Italy; 10 Clinical Unit of Neurology, Department of Medicine, Surgery, and Health Sciences, University of Trieste, Trieste, Italy; 11 Department of Engineering and Architecture, University of Trieste, Trieste, Italy

**Keywords:** Parkinson’s disease (PD), Mobile Brain/Body Imaging (MoBI), dual-tasking, neuromuscular function, older adults

## Abstract

**Background:** Neuromuscular dysfunction is common in older adults and more pronounced in neurodegenerative diseases. In Parkinson's disease (PD), a complex set of factors often prevents the effective performance of activities of daily living that require intact and simultaneous performance of the motor and cognitive tasks.

**Methods:** The cross-sectional study includes a multifactorial mixed-measure design. Between-subject factor grouping the sample will be Parkinson’s Disease (early PD vs. healthy). The within-subject factors will be the task complexity (single- vs. dual-task) in each motor activity, i.e., overground walking, semi-tandem stance, and isometric knee extension, and a walking condition (wide vs. narrow lane) will be implemented for the overground walking activity only. To study dual-task (DT) effects, in each motor activity participants will be given a secondary cognitive task, i.e., a visual discrimination task for the overground walking, an attention task for the semi-tandem, and mental arithmetic for the isometric extension. Analyses of DT effects and underlying neuronal correlates will focus on both gait and cognitive performance where applicable. Based on an a priori sample size calculation, a total N = 42 older adults (55-75 years) will be recruited. Disease-specific changes such as laterality in motor unit behavior and cortical control of movement will be studied with high-density surface electromyography and electroencephalography during static and dynamic motor activities, together with whole-body kinematics.

**Discussion:** This study will be one of the first to holistically address early PD neurophysiological and neuromuscular patterns in an ecologically valid environment under cognitive-motor DT conditions of different complexities. The outcomes of the study aim to identify the biomarker for early PD either at the electrophysiological, muscular or kinematic level or in the communication between these systems.

**Clinical Trial Registration:** ClinicalTrials.Gov, NCT05477654. This study was approved by the Medical Ethical Committee (106/2021).

## Introduction

Parkinson’s disease (PD) is the second most common neurodegenerative disease after Alzheimer’s disease, affecting 2–3% of the population over 65 years of age worldwide ^1, 2^. This progressive disorder results from the depletion of dopaminergic neurons in the substantia nigra pars compacta ^3^. In the early stages of the disease, PD patients present mainly with motor symptoms such as tremor at rest, rigidity, bradykinesia, postural instability, and gait disturbances, which become more complex in advanced stages and are accompanied by non-motor symptoms ^4^. Clinically, the most striking features of patients with postural instability/gait disorder (PIGD) are postural instability with falls and freezing of gait (FOG), which occur mainly in advanced stages and are episodic and unpredictable ^5, 6^. Gait impairments in PD patients encompass reduced speed and stride length, increased double limb support time, and stride-to-stride variability ^7– 10^.

Although PD motor symptoms dominate clinical presentations, most patients also experience cognitive and behavioral impairments, cognitive executive deficits, and depression ^11, 12^. The cognitive dysfunction observed in PD patients is primarily due to dopaminergic and cholinergic dysfunction ^13^, and is typically associated with impaired frontostriatal circuitry ^11, 14^, which impairs action planning and problem-solving ^14^, attentional set-shifting ^15^, response inhibition ^16^, and decision making ^17^. The three nonmotor frontostriatal circuits that connect frontal cortical regions to subcortical regions such as the basal ganglia originate in the dorsolateral prefrontal cortex (DLPFC), anterior cingulate cortex (ACC), and orbitofrontal cortex (OFC) ^18^.

A link between global cognitive dysfunction and motor symptoms in PIGD patients has been previously suggested ^18^. Compared to tremor-dominant patients (TD), patients with PIGD have greater impairments on measures of global cognition ^19^, a higher frequency of mild cognitive impairment ^20^, and an increased risk of developing dementia ^21^. In their study of 783 participants, Kelly and colleagues ^19^ found an association between deficits in global cognition, executive function, memory, phonemic fluency, and more severe symptoms of postural instability/gait disturbance. While visuospatial impairments were associated only with more severe freezing and poorer memory function was associated only with greater postural instability, executive function deficits were associated with greater postural instability, more severe freezing, and gait impairments. Interestingly, gait characteristics of speed, variability, and postural control were also found to precede and predict declines in attention and visual memory ^22, 23^, as well as dementia in old age ^24^.

To understand the complexity of PIGD, locomotion control has been studied extensively. To efficiently perform a locomotor task, one should be able to (i) generate proper force for the step, (ii) maintain postural balance, and (iii) continuously adapt to environmental demands and behavioral goals ^25^. To ensure optimal walking, multiple sensorimotor processes must be functionally integrated on several levels to maintain the forward propulsion of the centre of mass while maintaining dynamic stability ^26^. At the highest level, the timing of muscle contraction between agonists, antagonists, and synergists acting on both sides of the body are precisely regulated ^26^. At lower levels, the behavior of motor units (MU) pattern is regulated following a size principle to ensure smooth muscle contractions ^27^. However, sensorimotor processes are impaired in PD patients, resulting in altered motor activity patterns during rest and locomotion ^26^. Moreover, it has been shown that the simultaneous performance of cognitive and motor tasks can increase differences in motor performance between healthy individuals and PD patients ^26^.

In daily life, balancing or walking is rarely the only task performed at a given moment (single task; ST). Most often, it is performed together with another task (dual task; DT) or sometimes even several other tasks as a multitasking activity, resulting in competition for limited resources which can degrade performance on one or all tasks ^28^. In case of performing cognitive and motor tasks simultaneously, the performance decrements are a consequence of cognitive-motor interference (CMI). By comparing the performance success of both, ST and DT conditions, we can calculate dual-task costs (DTC) using the following formula: *(DT performance – ST performance) / ST performance* ^29– 32^. In PD patients, performing a cognitive serial subtraction task has been shown to impair the concurrently performed motor task as observed in decreased accuracy of force tracking ^33^, and promote deteriorating effects on the motor system such as inducing tremor ^34^. Studies of postural stability have shown that individuals with PD have impaired postural control under dual-task conditions ^35, 36^. Impaired walking in PD patients is more pronounced under cognitive-motor DT conditions ^37– 42^, with additional impairments observed in symmetry and coordination between the left and right steps ^43, 44^. Walking difficulties in PD patients can additionally be exacerbated in situations where the walking path is being restricted, such as narrow pathways/corridors/doorways or when walking between objects ^45^. For example, Cowie and colleagues ^46^ have shown that PD patients slow down at doorways to an extent that is inversely proportional to doorway width and that the frequency of freeze events increases in narrower doorways.

Another important aspect is the cognitive effort involved in activities of daily living, which varies according to the complexity of the situation and the associated cognitive demands ^47^. Regardless of complexity, cognitive processing is highly dependent on visual information, ranging from the simplest scenario, such as recognizing and responding to a red light, to a highly complex scenario involving navigating through an unfamiliar city on uneven surfaces while ensuring the safety of the accompanying infant. These situations illustrate the importance of visual information processing for the generation, execution, and adaptation of motor commands during walking ^48– 50^.

**Figure 1.**
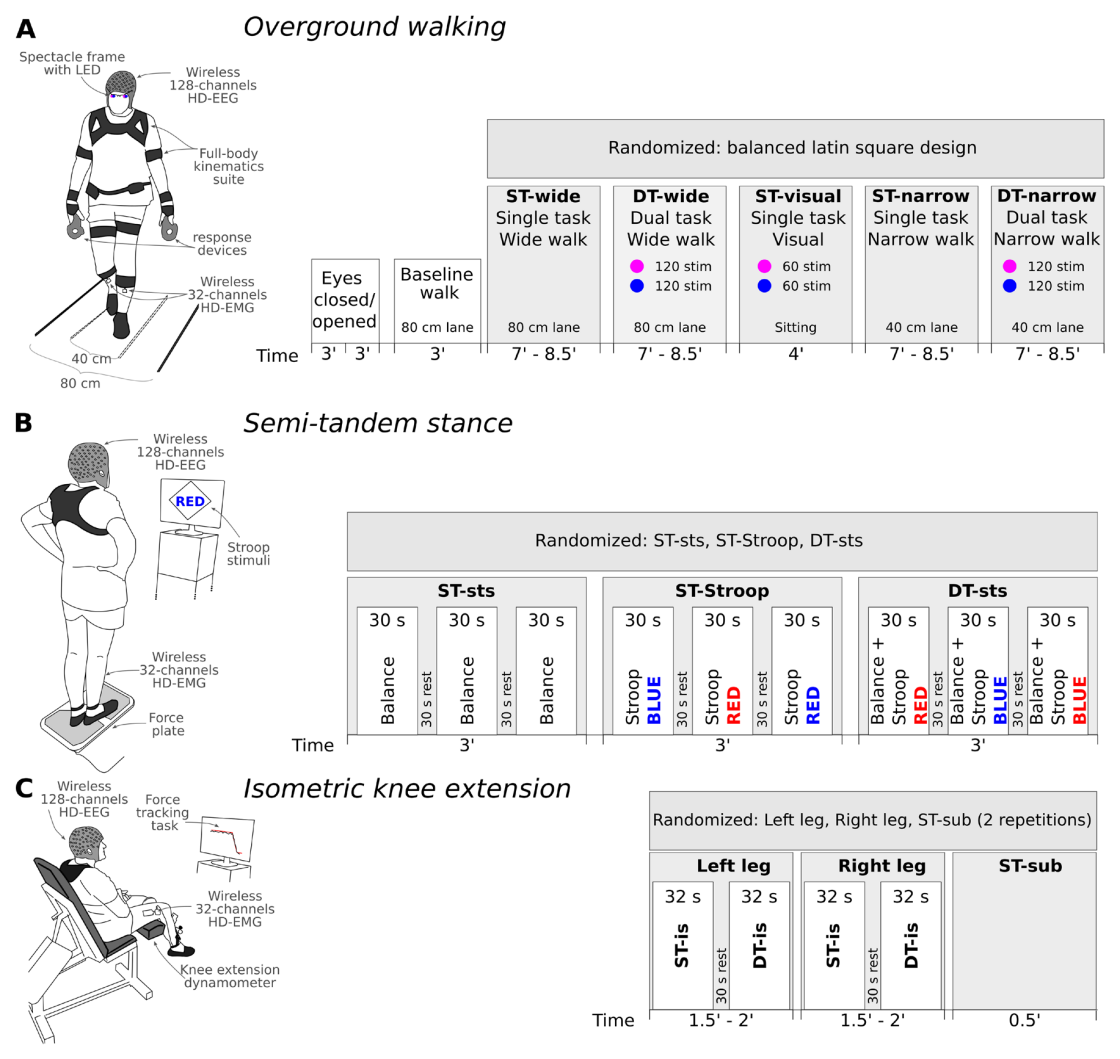
Graphic depiction of each motor activity, timeline of the procedure, and randomization order. ( **A**) Overground walking will start with a resting-state EEG recording with both eyes closed and opened, followed by a baseline walk, and finally randomized order of ST and DT conditions during overground walking on either wide or narrow lanes with/or visual discrimination task (blue and purple light). ( **B**) Next, the semi-tandem stance will be performed, again using randomization of the ST and DT conditions across participants, while the 30-second segments within each condition will be kept at fixed order. ( **C**) Lastly, during the isometric knee contraction the order of the leg blocks and ST-sub will be randomized, while during each leg block, the ST will always be performed before the DT. Duration of each recording is denoted below the time axis. For a detailed explanation refer to the main text. Abbreviations: ST – single task, DT – dual task, sts – Semi-tandem stance, sub – subtraction or mental arithmetic task.

PD patients experience walking difficulties and gait disturbances under the ST walking condition, which become further exacerbated under the DT condition. Despite many efforts, it remains unclear how motor and cognitive symptoms contribute to ST or DT walking difficulties ^29^. Investigating the interaction between motor and cognitive symptoms at behavioral and neurophysiological levels in ecologically valid settings could greatly enhance our understanding of the underlying mechanism of ST and DT performance decline. In the proposed study, we aim to apply a multilevel approach to understanding and investigating cognitive-motor interactions, with particular attention to the three motor activities characterized by different levels of cognitive-motor resource demands (see Figure 1). Specifically, the overground walking activity on a wide and a narrow path (ST-wide and ST-narrow) will constitute the core part of the study and will also be examined during a concurrent cognitive color discrimination task (DT-wide and DT-narrow); the cognitive task will additionally be examined in an ST condition for a comprehensive comparison (ST-visual). Second, postural control will be examined in the semi-tandem stance alone (ST-sts) and simultaneously with a cognitive task examining attention (DT-sts). The attention task will also be examined as ST in the seated position (ST-Stroop). Finally, in the seated position, isometric force development activity over time will again be examined alone (ST-is), under dual-task cognitive conditions while performing a series-3 subtraction task (DT-is), with the series-3 subtraction also performed in the absence of the motor task (ST-sub). This will allow systematic assessment and identification of motor and cognitive factors contributing to the symptomatology of PD patients. We are aware that the comparison between the DT conditions of the three motor activities is complicated by the use of different cognitive tasks, yet this decision was motivated to avoid extensive learning effects and increasing frustration during the measurement day.

Investigations of functional performance in more ecologically valid environments have recently been enabled by the Mobile Brain/Body Imaging (MoBI; ^51– 53^) approach. Here, MoBI will be used to investigate time-synchronized electroencephalographic (EEG) and electromyographic (EMG) recordings as well as body movements and task performance. Thus, the aim of the present study is to investigate the early PD disease-specific changes and laterality of neuronal rhythms during overground walking (dynamic condition), semi-tandem stance (standing static condition), and sustained isometric contraction (seated static condition), as well as under the ST and DT conditions. Compared with healthy participants, we hypothesize that early PD will have poorer ST performance on cognitive and motor tasks and increased DT costs on all motor activities. Furthermore, we expect that performance impairments with increased neural recruitment will be most pronounced in the highest motor complexity condition (walking in the narrow pathway) and even more pronounced in the corresponding DT variant (DT -narrow). 

## Methods

### Trial design

This protocol is formulated according to the SPIRIT (Standard Protocol Items: Recommendations for Interventional Trials) statement ^54^. It describes a multifactorial mixed-measure design and encompasses three different motor activities. In each motor activity, namely the overground walking, semi-tandem stance, and isometric contractions, one between-subject factor *PD* (early PD vs. neurotypical older adults) and within-subject factor *task complexity* (ST vs. DT) will be considered. In addition, in the walking activity, the second within-subject factor *walking difficulty* (wide vs. narrow) will be implemented. This leads to a mixed between-within 2 ( *PD*) x 2 ( *task complexity*) study design for the isometric and stance activities and to a mixed between-within 2 ( *PD*) x 2 ( *task complexity*) x 2 ( *walking difficulty*) design for walking activity. Figure 1 provides an overview of the study structure and timeline, as well as motor activities with ST and DT conditions embedded. A detailed description of the cognitive tasks and their respective stimuli can be found below under “Cognitive task performance”.

***Ethical approval and confidentiality*.** The study was registered at IRB of Trieste University Hospital – ASUGI, Trieste, Italy (ASUGI protocol number: 106/2021; approved 20.12.2022) and on ClinicalTrials.Gov under code: NCT05477654. The written informed consent for the patients will be obtained by a medical doctor (PM or MC), a movement disorder specialist, employed by the Neurological Clinic of Trieste University Hospital, while for the healthy participants the lead researcher will obtain it. All participant information and data will be stored securely and identified by a coded ID number only to maintain participants’ confidentiality.

***Managing adverse effect*.** The protocol and proposed experiments were modified after the pilot trials to minimize the occurrence of factors that led to experiment termination. Specifically, the number of stimuli and the duration of conditions were kept low enough to avoid fatigue but high enough to ensure adequate data quality, while the breaks between tasks/conditions were made sufficiently long. If reasons such as fatigue will nevertheless lead to a request for an interruption, the investigation will be terminated. Similarly, if patients/participants will experience dizziness or will experience adverse effects for other reasons, the examination will be terminated. A physician will be present to ensure physical safety. The experimental day is organized in three experiments, so the recorded data will provide valuable insights despite the number of experiments completed.

### Eligibility criteria

Inclusion criteria for the healthy older participants will be: (1) no diagnosis of cognitive or movement disorder, (2) Montreal Cognitive Assessment (MoCA; ^55^) score ≥ 24 points (3) Short Physical Performance Battery (SPPB) ^56^ score > 7 points (4) living independently in the community, (5) age range between 50 and 75 years, (6) no color blindness.

Inclusion criteria for the early-stage PD patients: (1) diagnosed according to the last International and Movement Disorder Society (MDS) criteria (2015), (2) duration of the disease less than 5 years, (3) positive Dopamine Transporter Scan (DATSCAN), (4) diagnosed akinetic-rigid or mixed phenotype, (5) score of 1 or maximally 2 on the Hoehn and Yahr scale ^57^, (6) MoCA score ≥ 24 points, (7) SPPB score > 7 points, (8) age range between 50 and 75 years, (9) no color blindness, (10) living independently in the community.

Exclusion criteria will be (1) advanced stages of PD (Hoehn and Yahr score > 2 points), (2) any acute or untreated chronic diseases, especially of the peripheral and central nervous system, such as heart failure, respiratory failure, severe osteoarthritis, and major psychiatric illness, such as depression, (3) MoCA score < 24 points, (4) severe disabling tremor, (5) recurrent falls, (6) impaired vision that is not corrected with e.g., glasses, (7) SPPB score ≤ 7 points.

### Outcome measures

This protocol focuses on the behavioral, cognitive, motor, and/or kinematic outcomes as well as neurophysiological markers associated with CMI during dual-tasking in healthy aged individuals and early PD patients. The accompanying information regarding the participants' demographics and general health will be treated as a secondary outcome and used to control for the confounds.

***Primary outcomes*.** The primary outcomes suggested by this protocol will be the DT effects associated with the concurrent execution of overground walking/semi-tandem stance/isometric contraction *and* a respective secondary cognitive task. The motor task parameters, e.g., the gait parameters using full-body kinematics (step length and width, stride time variability, walking speed), the center of pressure (COP) displacement for the static stance, and deviation of the required force development over time for the isometric contractions, will be investigated in the ST and DT conditions. Additionally, each cognitive task performance will be recorded in the ST condition to allow for the investigation of the DT effect on the cognitive level too. The cognitive ST condition will be performed in a seated position for all cognitive tasks (visual discrimination task, counting of Stroop stimuli, serial-3 subtraction; for description see below). High-density surface electromyography (HD-EMG) and high-density electroencephalography (HD-EEG) will be recorded in all motor activities in both ST and DT conditions. In the overground walking experiment, the DT condition will be created using a visual discrimination task, in the semi-tandem stance experiment the cognitive secondary task will be counting the occurrences of specific visual Stroop stimuli, and finally, in the isometric contraction experiment, the secondary task will encompass mental arithmetic. All tasks include aspects of executive functions. However, to avoid learning effects the task-set up was conducted differently between all conditions. Below are detailed descriptions of the cognitive and motor tasks. 

*Cognitive task performance:*

- Contra- vs. Ipsi-Lateral Visual Discrimination Task performance in walking activity: The outcome measures for a visual discrimination task will be response accuracy and response time. The implementation of such a task will allow for the recording of the event-related potentials as well as continuous brain activity. Stimuli of the cognitive task used in the ST and DT walking experiment will vary according to the presentation side (left vs. right), spectral properties (magenta vs. cyan), and presentation-response laterality (ipsi- vs. contra-lateral). DT costs and the associated neural underpinnings will be assessed in the cognitive task as well as in gait parameters.
- Silent counting of specific Stroop stimulus occurrences in the semi-tandem stance activity: Computerized Stroop task words “Blue” and “Red” will be presented one at a time in either blue or red ink color. Presenting stimuli at a rate of 1/s, participants will have to count the number of specific occurrences, e.g., “Blue” in blue ink, from the four possible options across 30-second intervals. This will be repeated three times, each time focusing on and counting a different color combination of stimuli.
- Mental arithmetic: Sequential subtracting is typically used as a screening procedure to assess attention/working memory functions. In our study, participants will be asked to quietly perform serial-3 subtraction from a random 3-digit number and verbally indicate the resulting number after a 30-second interval. This will be performed 3 times. The resulting number will be noted and will serve as an indirect measure of success. Standardization and adult norms are available ^58^.

*Motor performance:*

- For the walking experiment, the full-body kinematics will be captured using 17 inertial Shadow Motion sensors (Motion Workshop, Seattle, WA, USA). To ensure higher precision of body position and alignment correction in space, an additional infra-red based tracker (VivePro, HTC, Taoyuan, TW) surrounded by three base stations will be used. The following gait parameters will be extracted from the feet sensors: walking speed, step length, step width, double support time, and step-to-step variability. The arm swing and hip rotation information will be extracted using the arm and torso sensors.
- Second, postural control will be measured with a force plate (AMTI HE600600-2 k, Advanced Mechanical Technology, Inc., Watertown, MA, USA). The COP displacement in antero-posterior and medio-lateral directions will be calculated in cm.
- Lastly in the sitting activity of isometric knee extension, the ST and DT performance will be investigated using the isometric dynamometer (Wise Technologies Ltd., Ljubljana, Slovenia) *Muscle activity:*

EMG activity will be recorded using two wireless 32-channel probes (MUOVI, OT Bioelettronica S.r.l., Torino, Italy). For walking and semi-tandem stance, 8 x 4 matrix electrodes (10 mm interelectrode distance, HD10MM0804, OT Bioelettronica S.r.l., Torino, Italy) will be attached to bilateral tibialis anterior muscles. For the force tracking task, the electrodes will be attached to the bilateral vastus lateralis muscles. The electrodes and probes will be taped on the skin to minimize motion artifacts.

HD-EMG signals collected during walking and semi-tandem stance will be analyzed in the time and frequency domain using HD-EMG amplitude envelopes techniques to calculate entropy, HD-EMG amplitude barycenter, and spatial differences in HD-EMG signals. HD-EMG signals collected during the isometric force tracking will be decomposed in contributions of single MUs using blind source separation methods ^59^ to study individual motor unit firing patterns and early assessment of tremor.

*Brain activity*

EEG activity will be recorded using a mobile 128-electrode wireless system (CGX, Cognionics Inc., San Diego, USA). The electrodes will be mounted onto an elastic cap (easyCAP) and positioned according to the standard 5–10 system ^60^. Before recording brain activity during the three motor activities, the continuous resting-state EEG will be recorded at baseline while sitting; for 3 min with both eyes open and eyes closed. After this, the brain activity will be continuously recorded during ST and DT conditions in the overground walking, during postural semi-tandem stance, and finally for the static isometric knee extensions. The investigated event-related markers associated with visual processing in the visual discrimination task during walking activity and attentional task during the semi-tandem stance will be the visual P1, N1, and P2 components over the occipital cortex, and the P3 component over the parietal cortex assessed in both ST and DT conditions. The EEG data during all motor and motor-cognitive tasks will be analyzed by the means of time–frequency spectral analysis. The communication between the brain and muscles (i.e., cortico-muscular connectivity) will be assessed by EEG-EMG coherence analysis.

***Secondary outcomes*.** The secondary measures will be collected to assess the eligibility criteria and the general health status of cognitive and motor function. These measures will also be used to control for the covarying factors.

*Demographic questionnaire*s will collect data on participants' age, sex, ethnicity, body mass and height, leg length, and socio-educational status. Also, their handedness in 10 everyday situations will be asked.

The *general health questionnaire* will include PD-specific anamnestic questions for the PD patients, while in all participants we will scan for comorbidities, history and ongoing lower limb injuries/surgeries, presence of acute pains, sleeping hours of the last night, and coffee/energy drink intake on the measurement day. 

The *Short Falls-Efficacy-Scale-International* (SFES-I; ^61– 63^) is a 7-item questionnaire used to assess the fear of falling associated with the execution of easy and complex physical and social activities. A translated Italian version is available and we will provide a translation for the Slovenian-speaking subsample.

The *Short Physical Performance Battery* (SPPB; ^56^) will assess lower extremity function in the elderly and consists of three sections. First, the ability to perform a Romberg, semi-tandem, and tandem stance. Second, the time of walking a 4-meter track at a comfortable speed is measured. Third, participants must perform 5 sit-to-stand transfers as fast as possible, and time-to-completion is measured. Total SPPB score ranges from 0 (low mobility) to 12 (high mobility; up to 4 points per section) and takes up to 10 min to complete.

The *New Freezing of gait questionnaire* (N-FOG; ^64^) is a 9-item self-reported questionnaire measuring the freezing of gait and walking abilities in PD patients through various scenarios, and the impact of gait freezing on daily activities.

The *Falls History Questionnaire* (FHS) assesses the falls incidence, location, and consequence in the previous 12 months and is based on the recommendation of the Lamb and colleagues ^65^ and Lord and colleagues ^66^.

The *Movement Disorder Society Unified Parkinson’s Disease Rating Scale* (MDS-UPDRS; ^67^) is a comprehensive assessment of various aspects of PD including motor and non-motor experience of daily living and the related complications. The evaluation characterizes the extent and burden of the disease.

The *Montreal Cognitive Assessment* (MoCA; ^55^) is a one-page 30-item test developed for screening for mild cognitive impairment. It involves items to assess a range of cognitive domains, including executive functions, visuospatial abilities, language, attention, working memory, abstraction, and orientation to time and place. The duration of the assessment is about 10 min. The validated Italian ^68^ and Slovenian versions are available ^69^.

The *Trail Making Test* (TMT; ^70^) is a measure of psychomotor speed and attentional set-shifting. It requires the subject to connect 25 encircled numbers randomly arranged on a page in increasing order (Part A), and 25 encircled numbers and letters in alternating and increasing order (Part B) using a pen. Both part A and part B include practice exercises. About 5 to 10 minutes are needed for test administration. The outcome measure of the TMT is the time of completion.

The *Brief Mood Introspection Scale* (BMIS; ^71^) is comprised of 16 emotional/mood adjective or phrase items relating to the present moment and a participant is required to respond using a 4-point Likert scale ranging from definitely do not feel to definitely feel.

The *Beck’s Depression Inventory* (BDI; ^72^) consists of 21 items of depressive symptoms experienced over the past week. Each symptom is described in 4 increasing intensities, and a participant must decide which one best describes their state. Zero to maximum of 3 points are given per item proportionally to the chosen intensity.

The *Dual-Task Strategy Assessment* is a modified version of the tool used by Wollesen and colleagues ^73^ and consists of 6 closed-type questions/statements regarding the employed strategy and allocation of one's attention during DT performance.

The *NASA Total Load Index* (NASA-TLX; ^74^) is a tool assessing subjective workload across 6 subscales: mental, physical, and temporal demand, as well as performance, effort, and frustration. The participant marks the degree of workload on a visual scale, ranging between 1 – 21. The overall score is calculated as a sum or average of the 6 subscales and the questionnaire is applied after each randomized condition in the overground walking activity.

### Recruitment process

PD patients will be recruited at the Neurological Clinic of Trieste University Hospital, while the research will be conducted at the ASUGI Physiotherapy Gym of the Trieste University. During a regular visit to the Movement Disorders outpatient clinic, the potentially suitable candidates will be invited to participate and will have the study design, protocol as well as all procedures and treatment of clinical data fully explained, as for standard institutional practice. The control group of neurotypical older adults will be recruited via the Daily Activities Center Koper, a database of previously enrolled subjects in the experiments of the Institute of Kinesiology, Science and Research Centre Koper, and via a word of mouth. The initial telephone screening in healthy adults (demographic and general health screening) will be performed to identify the potential participants. The enrolled patients and healthy adults will undergo a comprehensive screening session including motor and neurocognitive assessment (Measurement Day 1 - M1). Having passed the inclusion/exclusion criteria confirmed on the M1, they will finally be invited to participate in Measurement Day 2 (M2). Participants will not be compensated for collaborating in the respective study in any way.

### Procedure, tasks, and stimuli

The study proposed by this protocol will consist of 2 measurement days per participant. Following the recruitment and signing of the informed consent, the participants’ characteristics will be collected, and the questionnaires and tests listed under the Secondary outcomes section performed (except for the Dual-Task Strategy Assessment and NASA-TLX). The M1 will last approximately 1–1.5 hours. After the completion of the first measurement day, the fulfillment of inclusion/exclusion criteria will be carefully inspected, and the fitting participants will be invited for the M2. These participants will be shown a recording of the M2 setting embedded within the actual environment with a fully equipped volunteer performing a DT walking condition. The recording will serve to familiarize the participants with the setting and address the potential anxiety of encountering new situations in a highly controlled environment. This will also be the opportunity to respond to participants’ questions and concerns.

The M2 will begin with the participant preparation; fixing EEG, HD-EMG, and full-body kinematic sensors on the participant. The order of motor activities, namely the overground walking, semi-tandem stance, and isometric force development, will remain fixed for all participants (see Figure 1). The measurement day 2 will last approximately 5 hours. Below we specify the procedure of each motor activity and the associated cognitive tasks in more detail.

***Overground walking*.** After the resting state EEG recording (3 min with eyes closed and 3 min with eyes open), the baseline gait performance will be assessed by participants walking at self-selected speed but without carrying the response controllers in their hands along the 80 cm wide and 8m long lane for 3 minutes (excluding turn time). Then, the order of the ST and DT conditions will be randomized using a balanced Latin square formula for the 5 conditions, namely the ST-wide, DT-wide, ST-narrow, DT-narrow, and ST-visual conditions. In the visual discrimination task, the stimulus-response combinations will either share the side or not – ipsilateral or contralateral stimulus-response combinations, respectively (see Table 1). A practice session will be conducted prior to each condition to ensure task comprehension and pacing familiarization.

**Table 1.** Stimuli number of the cognitive visual discrimination task separated by motor task condition, hemifield presentation side, cognitive task modality, visual modality-specific properties, and presentation-response compatibility (in yellow).

		COGNITIVE TASK: Visual discrimination		
MOTOR TASK:	Presentation side:	Magenta	Cyan	∑
**ST-visual (Sitting)**	Left	30	30	60
	Right	30	30	60
**DT-wide (Walking)**	Left	60	60	120
	Right	60	60	120
**DT-narrow (Walking)**	Left	60	60	120
	Right	60	60	120
	**∑**	300	300	**600**
Correct response is	contra-lateral		Ipsilateral to the presentation side	

The ST-visual condition will be performed in the seated position in the middle of the walking lane while wearing response devices - VIVE pro controllers. In addition, participants will carry a custom-made spectacle frame with attached LED light on each end covering the periphery of the visual field, which will serve to provide visual stimulation. A total of 240 stimuli (120 magenta and 120 cyan) will be presented on either the left or right spectacle side (50-50%; see Table 1 for stimuli characteristics). The interstimulus intervals will vary between 400–800 ms, and a response window will be limited to 900 ms after the stimulus presentation and indicated by pressing the button on the left- or right-hand held controller. The trial duration will vary between 1.4 to 1.8 s. Should no response be given within the predetermined response window, the next trial will start after 900 ms.

ST-wide and ST-narrow walking multimodal data will be collected while participants walk at self-selected speed in the wide (80 cm) and narrow (40 cm; ^75^) lane of 8 m length, respectively. The entire walking distance excluding the turn path will amount to 384 m (approximately 420 m including the turn path). Passing the end of the lane, a turn will follow, and a new gait pass will start upon re-entering the lane. Participants will be carrying response devices (VIVE pro controllers) although they will not be used in these two conditions. The estimated time for each condition is 7-8.5 minutes with turn times included.

In both DT-wide and DT-narrow walking conditions, the timing for visual stimuli presentation and responses will be the same as in the ST-visual condition. However, at the same time, participants will be walking along either a wide or narrow lane (described above) and upon reaching the end of the lane will be required to make a turn and re-enter the lane. The walking time of 7-8.5 minutes is estimated per condition (including the turn time). Per one lane length, 5 stimuli will be presented. The condition will end as soon as a participant walked 420 m (including the turn path) and 240 stimuli were presented.

Between each condition, a 2-min rest will be assured, during which NASA-TLX will be applied and participants will be asked to refer to the past condition. At the end of the 5 conditions of the walking activity, also the dual-task strategy assessment will be asked.

***Semi-tandem stance*.** Next, in semi-tandem postural experiment participants will perform three 30-second repetitions of semi-tandem stance for the ST-sts motor condition. This will be repeated in the DT-sts condition while simultaneously counting the occurrences of specific Stroop stimuli, and in the seated position for the ST-Stroop condition. The order of the ST and DT conditions will be counterbalanced across participants, however, the three 30-second repetitions of a certain condition will follow in succession in a fixed order.

Participants will be standing on a force plate (AMTI HE600600-2 k, Advanced Mechanical Technology, Inc., Watertown, MA, USA) in a semi-tandem position with their preferred (but always the same) foot in front and with their hands fixed on their hips. Over the 30-second runs in both ST-sts and DT-sts conditions, an average COP sway length (in cm) will be taken as an outcome measure. In DT-sts and ST-Stroop conditions the outcome measure will (also) be the reported number of the required observed Stroop-stimuli. In each of the three 30 sec repetitions, different combinations of Stroop stimuli will have to be attended, (e.g. “Blue” in blue ink, “Red” in red ink, and “Blue” in red ink). We choose silent counting of the Stroop-stimuli occurrences over loud to reduce the talking-related EEG artifacts. To motivate the participants into performing the cognitive task, they are required to report the result at the end of each 30-second trial. During the ST-sts condition, participants will be asked to direct their gaze to a fixation cross presented on the screen at the level of their eyes. 

***Isometric knee extension*.** Finally, for the trapezoidal isometric force tracking participants will undergo two 32-second force tracking sessions of knee extension (6 s rising phase, 20 s sustained phase, 6 s decline phase) per both right and left lower limbs in the ST-is motor condition. The participants will be sitting in a knee extension dynamometer (Wise technologies, Ljubljana, Slovenia) equipped with a force sensor and they will be instructed to actively contract their knee extensors to produce force up to 30% of their maximum voluntary contraction (MVC). The force tracking task will be repeated while concurrently performing the secondary serial-3 subtraction task quietly during the DT-is condition for both right and left lower limbs. The serial 3-subtraction task will also be quietly performed in an ST-sub condition for 32 seconds. Each time, a random 3-digit number between 300 and 500 will be selected as a starting point and at the end of the 32 second period, participants will be asked to report the number they had reached. The order of the right and left limbs and the ST-sub will be counterbalanced across the participants, while they will always perform the ST-is before the DT-is with a respective lower limb. The task will consist of 2 rounds of described conditions. 

***Randomization sequence*.** The randomization sequence will be created via the online balanced Latin square tool separately for each experiment.

### Data streaming and management

All data collected through paper-pencil tests and questionnaires will be digitized for further processing. Functional testing will be controlled through Unity3D (Unity Technologies, San Francisco, USA), which will also assign basic demographic data to each participant's measurement data. All functional measurements will be streamed and synchronized with the Lab Streaming Layer (Swartz Center for Computational Neuroscience, UCSD, USA). The synchronized data streams will comprise gait analysis (Motion Workshop, Seattle, WA, USA and VivePro, HTC, Taoyuan, TW), EEG (CGX, Cognionics Inc., San Diego, USA), and HD-EMG (OT Bioelettronica S.r.l., Torino, Italy) recordings, and secondary task performance (VivePro, HTC, Taoyuan, TW & Raspberry Pi, Raspberry Pi Foundation, Cambridge, UK). Day-to-day management of the study, including data management, will be coordinated by the principal investigator of the study. All data collected will be fully anonymized. Each participant will be assigned a unique study identification number. Access to the data will be restricted to TwinBrain researchers and staff, in possession of authorization rights. Directly identifiable data (e.g., name, address, phone number) are kept secure and separate from other study data. Data procedures will be in accordance with the General Data Protection Regulation (GDPR). In accordance with the Ethics Committee commitment, we will obtain informed consent from participants to share their anonymous data with other researchers. Data management will be regularly reviewed by an internal data monitoring committee composed of data experts, engineers, psychologists, and movement disorder physicians.

### Statistical analysis and power calculation

Descriptive data will be presented as means (M) and standard deviations (SD). The homogeneity of variances and normality of distribution of parameters will be tested with Levene's test and Shapiro-Wilk test, respectively. Using univariate analyses of variances (ANOVA), the between-subject factor grouping the sample will be *PD* (early PD vs. no PD), while the within-subject factors will be the *task complexity* (ST vs. DT) in each motor activity, i.e., the overground walking, semi-tandem stance, and isometric force knee extension, as well as the *walking condition* (wide vs. narrow) for the overground walking only. Significance will be set at alpha = 0.05. The effect size will be presented as the unbiased adjusted partial eta squared ^76^. All post-hoc comparisons will control for multiple comparisons (e.g., Bonferroni). Statistical analyses will be done using SPSS (IBM, SPSS Inc, Chicago, IL, United States) or R (R Core Team, 2013).

Sample size estimation calculated using g*Power software (a priori: F-tests; ANOVA: repeated measures, within-between interaction; effect size f = 0.2; alpha error probability = 0.05; power (1-beta error probability) = 0.8; number of groups = 2; number of measurements = 3) revealed a total of *N* = 42 subjects, half of who will be healthy older adults, and the other half will belong to early PD patient population.

## Discussion

Recent advances in neuroimaging, wearables and wireless technologies offer a range of opportunities to better understand human behavior and link it to cortical and muscular activation patterns. MoBI enables the investigation of brain and muscle dynamics in conjunction with biomechanical parameters and secondary sensorimotor processes. The lack of scientific evidence in clinical populations has prompted us to conduct the present study with early-diagnosed PD patients. Therefore, the aim of this study is to investigate the differences in functional static and dynamic performance between healthy and age-matched early-PD populations. The effect of interest will be investigated through DT effects and associated neuromuscular underpinnings. Specifically, using the MoBI approach, EEG, HD-EMG, and whole-body kinematics will be recorded under various ST and DT conditions to uncover the underlying mechanisms of force production, balance, and gait control in early-diagnosed PD patients. The underlying processes of interaction between motor and cognitive tasks will be identified at behavioral and neurophysiological levels under conditions of varying complexity. The general hypothesis is that there are performance differences and corresponding neuromuscular correlates between subgroups, with the highest neuromuscular compensations occurring in the PD group. We hypothesize that the performance differences are related to the different motor-cognitive processes such as stimulus input, resource allocation, and movement execution. Our goal is to find a neuromuscular biomarker that distinguishes the two populations and thus reflects an early onset of neuromuscular deficit in PD.

We anticipate several challenges in conducting this study. First, recruitment of early-stage PD will be a relatively lengthy process because of the limited number of such patients in the Trieste region (Italy). Second, the complexity of the test blocks is planned in such a way that subjects will have enough breaks and will be able to rest between trials. However, the long protocol (about 5 hours) will require careful preparation and handling with constant medical monitoring. We will try to maintain motivation throughout the measurement and avoid fatigue by taking multiple breaks. Finally, in our sample of early PD, we do not expect the occurrence of FOG during our measurements. A hypothetical explanation for the occurrence of FOG is that the narrowness of the corridor causes an increase in optic flow, as the visual image of near objects moves faster on the retina than that of more distant objects ^77^. Thus, visual and vestibular signals provide conflicting information to the brain in such situations. In our case, participants will be walking in a wide and narrow lane that gives the impression of a narrower path but does not enforce the narrow space/corridor.

In summary, the current study employs a state-of-the-art methodology to assess neuromuscular outcomes that aim to elucidate important underlying mechanisms of neural control of movement during dual motor-cognitive tasks in healthy older adults and in patients with early-diagnosed PD. Thus, we aim to uncover an early biomarker of movement (in)efficiency.

### Dissemination

The communication about the running, upcoming or past activities for the general and specific public are regularly uploaded to the webpage of the TwinBrain project ( www.twinbrain.si). In addition, the webpage and social media contain links to all media press, podcast series, and newsletters which are intended to reach a wide audience.

The scientific output of the study will primarily target researchers and medical doctors. The output of the proposed protocol will produce at least three scientific papers, which will be submitted to peer-review scientific journals. In addition, the results of the clinical trial will be disseminated at content-related conferences, seminars, and congresses, such as the International Congress of Parkinson’s Disease and Movement Disorders and the Mobile Brain/Body Imaging Conference. 

The website will also be used for the long-term sustainability of the dissemination which will continue to communicate research findings and ongoing updates, and partnering with relevant organizations to continue research and dissemination efforts.

### Study status

The protocol has been tested on 5 pilot healthy young subjects, intended to detect potential issues with the software and recorded data. Upon resolving all the issues, three healthy older adults underwent the protocol to assess subjective fatiguing effects in the target age group and to identify the strategies to ameliorate the fatigue. Currently, 6 subjects had been tested, of which 4 belonged to the health group and 2 were PD patients.

### Ethics and consent

The study will be conducted in accordance with the ethical standards of the 1964 Declaration of Helsinki and the guidelines of Good Clinical Practice. IRB of Trieste University Hospital – ASUGI, Trieste, Italy approved this study protocol (ASUGI protocol number: 106/2021). The study was registered on ClinicalTrials.Gov under code: NCT05477654. The informed consent for each study participant will be obtained by a medical doctor (MC or PM), a movement disorder specialist, employed by the Neurological Clinic Trieste. All participant information and data will be stored securely and identified by a coded ID number only to maintain participants’ confidentiality.

## Data Availability

No underlying data are associated with this article. Zenodo: Demographic and General Health Questionnaire. https://doi.org/10.5281/zenodo.7768228 ^78^ Data are available under the terms of the Creative Commons Attribution 4.0 International license (CC-BY 4.0). UM, KG, CM, and PM conceived of the study and are grant holders. UM with help of MP, MMŠ, and MK drafted the manuscript. BW supervised and validated the DT settings All authors contributed to the refinement of the study protocol and approved the final manuscript.
