# Supplementary material for: Neuromuscular assessment of force development, postural, and gait performance under cognitive-motor dual-tasking in healthy older adults and people with early Parkinson's disease: Study protocol for a cross-sectional Mobile Brain/Body Imaging (MoBI) study
Source: Open Res Eur. 2023 Nov 10;3:58. Originally published 2023 Apr 17. [Version 3] doi: 10.12688/openreseurope.15781.3 (PMC10674089; doi:10.12688/openreseurope.15781.3)
Supplement: Peer review history

# Reviewer report: Reviewer response for version 3

**DOI:** 10.21956/openreseurope.18081.r36176

**Published:** 2023-11-24

**Authors:** Nicola J Ray

**Affiliations:** 1 Manchester Metropolitan University, Manchester, England, UK

I believe the authors have now addressed all my concerns.

Is the study design appropriate for the research question?

Partly

Is the rationale for, and objectives of, the study clearly described?

Partly

Are sufficient details of the methods provided to allow replication by others?

No

Are the datasets clearly presented in a useable and accessible format?

Not applicable

Reviewer Expertise:

NA

I confirm that I have read this submission and believe that I have an appropriate level of expertise to confirm that it is of an acceptable scientific standard.

# Reviewer report: Reviewer response for version 2

**DOI:** 10.21956/openreseurope.17851.r34667

**Published:** 2023-09-08

**Authors:** Nicola J Ray

**Affiliations:** 1 Manchester Metropolitan University, Manchester, England, UK

The authors have much improved the organisation of their proposal. I still have some comments:

 Minor: Primary and secondary outcomes has a special meaning that in clinical research, and the term is used incorrectly here. Primary/secondary study outcomes are those that arise from any primary/secondary study aim. Rather, in the current proposal, the authors have listed their full testing battery under Primary and Secondary outcomes. 

 Minor: Reviewer 2 suggested not to use 'Patients' to describe your 'Participants'. I agree with this and suggest this wording is changed throughout. 

 Minor: In my previous report I identified an issue with the screening procedure. There remains an issue with this: 

 Inclusion criteria are PD diagnosis, PD duration, Dopamine Transporter Scan (DATSCAN), diagnosed akinetic-rigid or mixed phenotype, Hoehn and Yahr scale, MoCA , SPPB score, age, no color blindness, independent living. Additional exclusions are (3) MoCA score < 24 points, (4) severe disabling tremor, (5) recurrent falls, impaired vision.

 The first testing session lasts 1.5 hours. Does this mean more tests are planned than are included in the exclusion criteria? This would mean that ineligible participants are taking part in research elements of the study. Will the authors discard this data? It is ethically dubious to ask a person with Parkinson's to take part in a 1.5 hour testing session for a study that their data cannot contribute to. Many of the tests included in their inclusion criteria are likely done routinely in the clinic? Additionally, the questionnaires are all self-report, and could be sent to people's homes before asking them to come in for testing?

 I understand the testing needs to take place over two visits since the battery is extensive, but screening is usually done first and no tests that do not contribute to screening should be completed in participants who are not eligible. If I have misunderstood the proposal, and only screening tests will be done on M1, I suggest the proposal lists explicitly 'M1 testing battery' and 'M2 testing battery'.

Is the study design appropriate for the research question?

Partly

Is the rationale for, and objectives of, the study clearly described?

Partly

Are sufficient details of the methods provided to allow replication by others?

No

Are the datasets clearly presented in a useable and accessible format?

Not applicable

Reviewer Expertise:

Parkinson's disease

I confirm that I have read this submission and believe that I have an appropriate level of expertise to confirm that it is of an acceptable scientific standard, however I have significant reservations, as outlined above.

# Response

**Published:** 2023-10-12

**Authors:** Uros Marusic

**Affiliations:** Science and research centre Koper, Slovenia

The authors have much improved the organisation of their proposal. I still have some comments:

 Minor: Primary and secondary outcomes has a special meaning that in clinical research, and the term is used incorrectly here. Primary/secondary study outcomes are those that arise from any primary/secondary study aim. Rather, in the current proposal, the authors have listed their full testing battery under Primary and Secondary outcomes. 

 ////////OUR RESPONSE////////Thank you for this comment. To make the phrasing less ambiguous concerning its alignment with the jargon of the clinical practice, we changed the *primary* and *secondary* outcomes to *principal* and *supporting* outcomes, respectively. In fact, by doing so we believe the description now fits the contents to a greater degree.

 Minor: Reviewer 2 suggested not to use 'Patients' to describe your 'Participants'. I agree with this and suggest this wording is changed throughout. 

 ////////OUR RESPONSE////////We agree with the suggestion and have changed the wording to *people with PD* to generally refer to PD patients and *participants *when we are addressing specifically the people involved in the study. 

 Minor: In my previous report I identified an issue with the screening procedure. There remains an issue with this: 

 Inclusion criteria are PD diagnosis, PD duration, Dopamine Transporter Scan (DATSCAN), diagnosed akinetic-rigid or mixed phenotype, Hoehn and Yahr scale, MoCA , SPPB score, age, no color blindness, independent living. Additional exclusions are (3) MoCA score < 24 points, (4) severe disabling tremor, (5) recurrent falls, impaired vision.

 The first testing session lasts 1.5 hours. Does this mean more tests are planned than are included in the exclusion criteria? This would mean that ineligible participants are taking part in research elements of the study. Will the authors discard this data? It is ethically dubious to ask a person with Parkinson's to take part in a 1.5 hour testing session for a study that their data cannot contribute to. Many of the tests included in their inclusion criteria are likely done routinely in the clinic? Additionally, the questionnaires are all self-report, and could be sent to people's homes before asking them to come in for testing?

 I understand the testing needs to take place over two visits since the battery is extensive, but screening is usually done first and no tests that do not contribute to screening should be completed in participants who are not eligible. If I have misunderstood the proposal, and only screening tests will be done on M1, I suggest the proposal lists explicitly 'M1 testing battery' and 'M2 testing battery'.

 ////////OUR RESPONSE////////Thank you for pointing this issue out. Upon reviewing the text we agreed that the description of the procedure is not completely clear. In hopes of bringing more clarity as to what, how, and in what order the recruitment activities were performed, we made some adaptations to the text under *Recruitment process* and *Procedure, tasks, and stimuli* sections.

# Reviewer report: Reviewer response for version 1

**DOI:** 10.21956/openreseurope.17047.r32804

**Published:** 2023-07-10

**Authors:** Nicola J Ray

**Affiliations:** 1 Manchester Metropolitan University, Manchester, England, UK

The authors propose to use a MoBI approach to investigate cognitive and motor interactions in People with Parkinson's compared with controls. Participants will perform motor tasks under ST and DT conditions. 

 I believe the presentation of this study requires closer attention. There are methods presented in the intro, for example. The description of the primary outcomes are very vague, and the hypotheses presented would only confirm findings already known in the literature. What does the MoBI approach add, and how will the authors analyse the data in order to add new knowledge to the field.

 The study design also requires closer attention. The different cognitive tasks will present a larger problem than acknowledged in comparing across tasks. I suggest to use a well practised task for all. 

 Additionally, some of these tasks will interfere with ability to perform the motor tasks for reasons not related to CMI. For example, requiring people perform a visual task during walking will change their ability to use visual information for the walking task itself. This will deteriorate performance during DT conditions, but not because of cognitive interference.

 There also seems to be two levels of eligibility - one for M1 and the other for M2. However no information is provided to understand why this is.

Is the study design appropriate for the research question?

Partly

Is the rationale for, and objectives of, the study clearly described?

Partly

Are sufficient details of the methods provided to allow replication by others?

No

Are the datasets clearly presented in a useable and accessible format?

Not applicable

Reviewer Expertise:

Parkinson's disease

I confirm that I have read this submission and believe that I have an appropriate level of expertise to state that I do not consider it to be of an acceptable scientific standard, for reasons outlined above.

# Response

**Published:** 2023-08-18

**Authors:** Uros Marusic

**Affiliations:** Science and research centre Koper, Slovenia

\\\\\\\\\\The authors propose to use a MoBI approach to investigate cognitive and motor interactions in People with Parkinson's compared with controls. Participants will perform motor tasks under ST and DT conditions. 

 I believe the presentation of this study requires closer attention. There are methods presented in the intro, for example. The description of the primary outcomes are very vague, and the hypotheses presented would only confirm findings already known in the literature. What does the MoBI approach add, and how will the authors analyse the data in order to add new knowledge to the field. //////////

 Thank you for the remark. As suggested, we reorganized the introduction section and moved the methods-oriented parts to the methods. As stated under the *Data streaming and management* chapter, our recordings of the hdEEG, hdEMG, and full-body kinematics will be time-synchronized while a participant will perform an overground walking task. Time synchronization will allow us to investigate the brain-muscle cross-talk for the first time in such a population in addition to unraveling the physiological dynamics in each modality separately. Because the technology and the whole MoBI field are relatively new (Gramann et al., 2010, doi: 10.3389/fnhum.2010.00202) such cross-modal investigations hold promise to better understanding the fundamental surface physiology and their potential compensatory mechanisms in PD patients during tasks resembling everyday functioning. It has been shown that PD patients demonstrate a hypersynchrony in the beta oscillatory range which is related to bradykinesia (Jenkinson & Brown, 2011; doi:10.1016/j.tins.2011.09.00). We made modifications to the *Brain Activity* chapter, where we highlight the novel approach of performing a gait-phase modulations analysis (Seeber et al. 2014, doi: 10.3389/fnhum.2014.00485) that has not yet been performed in PD patients and would allow investigating how and when is beta modulated relative to the gait cycle. Due to its association with symptom manifestation, the outcomes of such analysis carry great functional significance.

 \\\\\\\\\\The study design also requires closer attention. The different cognitive tasks will present a larger problem than acknowledged in comparing across tasks. I suggest to use a well practised task for all.  //////////

 We appreciate you pointing out this aspect, as we spent quite some time discussing this issue ourselves. We wrote in the manuscript: » *We are aware that the comparison between the DT conditions of the three motor activities is complicated by the use of different cognitive tasks, yet this decision was motivated to avoid extensive learning effects and increasing frustration during the measurement day.« *We are fully aware that a direct comparison across tasks will not be possible, however after a thorough consideration we decided upon taking this tradeoff instead of having participants perform thousands of trial repetitions of a single task which would promote a massive learning effect and automaticity. Such experimental (non)manipulation would in addition mean a step away from the ecological validity, which is one of the primary intents in the MoBI field. Being aware of this, we never intended on comparing across motor activities, that is overground walking vs balancing vs isometric knee extensions, but rather within each motor activity, meaning single-task walking vs dual-task walking, for instance. The main comparison will always be performed within a certain motor activity experiment. Examining the performance across all motor activities will, however, allow for making general claims, such as cognitive-motor interference was/wasn't observed in the respective motor activity/ies. For the balance and isometric experiments, the cognitive tasks we decided to use were derived from our previous work (for instance: Marušič et al., 2019,  DOI: 10.1186/s11556-018-0209-7 ; Peskar et al. 2023, doi: 10.1016/j.biopsycho.2023.108543)  and our research interests to better understand how the executive function as a complex and advanced cognitive system support our voluntary actions. For the walking activity, the visual secondary task was used because visuo-motor coordination holds great ecological validity. However, because exploring visual processing while overground walking is rather challenging due to the movement itself and controlling for the constant stimulus distance, evidence in this cross-domain remains rather scarce. For this reason, we decided to use a relatively simple visual working memory task (visual discrimination) not to overload the visual system. We believe our findings will contribute significantly to this as-of-yet vastly unexplored field. [Please consider also the answer to your next comment below.] Additionally, one of our goals (which exceeds the present protocol) is to compare the walking data in PD patients and in patients with hearing impairment (see protocol Wunderlich et al 2021, doi: 10.3389/fnagi.2021.773287). Lastly, to avoid recording adaptation to the task, all tasks will be well-trained during the practice run prior to recording.

 \\\\\\\\\\Additionally, some of these tasks will interfere with ability to perform the motor tasks for reasons not related to CMI. For example, requiring people perform a visual task during walking will change their ability to use visual information for the walking task itself. This will deteriorate performance during DT conditions, but not because of cognitive interference. //////////

 » *Cognitive-motor interference (CMI) is evident when simultaneous performance of a cognitive task and a motor task results in deterioration in performance in one or both of the tasks, relative to performance of each task separately* (Plummer et al., 2013 (doi: 10.1016/j.apmr.2013.08.002).« According to this definition, CMI as a consequence of the limited attentional resources is precisely *the reason* why the visual task during walking would result in deteriorated gait performance. Not only it offers a theoretical model for understanding the mechanism behind performance decrements, but, in addition, tackles the functional capacities of PD patients in real-world situations. For instance, in reality, on a busy pavement surrounded by heavy traffic, the motor actions have to be continuously adapted with respect to the ever-changing environment. Our perception and appropriate reactions to the environment will ensure our safety (such as reacting to or withdrawing action in response to passing cars or traffic lights). Here, vision arguably demonstrates the paramount importance to guide the translations of perception to motor actions. The ability to retain independence in PD patients can be legitimately assessed using a visual cognitive-motor dual-task walking experiment. In practical terms, however, the spectacle frame with LED lights attached to each end was custom-made with the awareness to avoid the coverage of a significant portion of the visual field, from which important information about the environment (and safety) could be detected. Also, because the frame (together with the lights) is attached to a participant, they are free to move the head around in all directions and use all visual angles to uncover potential occlusion to the walking lane, if needed. When using MoBI, we are translating the overly-controlled laboratory settings to more ecologically valid conditions, and through these lenses, the proposed paradigm is at the frontier of what technology currently allows. The outcomes of the present study hold great translational promise. 

 \\\\\\\\\There also seems to be two levels of eligibility - one for M1 and the other for M2. However no information is provided to understand why this is. //////////

 Under the *Recruitment Process* we wrote » *The enrolled patients and healthy adults will undergo a comprehensive screening session including motor and neurocognitive assessment (Measurement Day 1 - M1). Having passed the inclusion/exclusion criteria confirmed on the M1, they will finally be invited to participate in Measurement Day 2 (M2).« *M1 constitutes the first measurement day during which all the secondary outcomes are collected; these outcomes at the same time confirm either the inclusion or exclusion criteria. Measurement day M2 then constitutes the 3 motor activity experiments in combination with hdEEG, hdEG, and full-body kinematics. If you suggest to add additional information to make the procedure clearer, we are happy to do so.

# Reviewer report: Reviewer response for version 1

**DOI:** 10.21956/openreseurope.17047.r31835

**Published:** 2023-06-13

**Authors:** Breiffni Leavy

**Affiliations:** 1 Department of Neurobiology, Karolinska Institutet, Huddinge, Sweden

This protocol describes the planned test conditions for a cross-sectional investigation of the early PD disease-specific changes in motor-cognitive performance, assessed during three test conditions, compared to healthy age-matched controls.

 Background and rationale are clearly outlined, relevant and in line with the current evidence in the field.

 Methods and test protocols are clearly outlined and described in detail with an ambitious and widely encompassing list of outcome measures. I cannot see anything lacking. 

 In summary, this is a clearly defined and scientifically robust plan to investigate motor-cognitive performance in mild PD.

 Suggestion: One minor semantic alteration I suggest to bring the test in line with use of a person-centered language is not to refer to 'PD patients' (occurs frequently in the text) but people with PD. People are most often not patients, and not in the context of  cross-sectional research.

Is the study design appropriate for the research question?

Yes

Is the rationale for, and objectives of, the study clearly described?

Yes

Are sufficient details of the methods provided to allow replication by others?

Yes

Are the datasets clearly presented in a useable and accessible format?

Not applicable

Reviewer Expertise:

Rehabilitation for Parkinson's disease

I confirm that I have read this submission and believe that I have an appropriate level of expertise to confirm that it is of an acceptable scientific standard.
